# FcγR Binding and ADCC Activity of Human IgG Allotypes

**DOI:** 10.3389/fimmu.2020.00740

**Published:** 2020-05-06

**Authors:** Steven W. de Taeye, Arthur E. H. Bentlage, Mirjam M. Mebius, Joyce I. Meesters, Suzanne Lissenberg-Thunnissen, David Falck, Thomas Sénard, Nima Salehi, Manfred Wuhrer, Janine Schuurman, Aran F. Labrijn, Theo Rispens, Gestur Vidarsson

**Affiliations:** ^1^Sanquin Research and Landsteiner Laboratory, Department of Immunopathology, Amsterdam UMC, University of Amsterdam, Amsterdam, Netherlands; ^2^Sanquin Research and Landsteiner Laboratory, Department of Experimental Immunohematology, Amsterdam UMC, University of Amsterdam, Amsterdam, Netherlands; ^3^Genmab, Utrecht, Netherlands; ^4^Center for Proteomics and Metabolomics, Leiden University Medical Center, Leiden, Netherlands

**Keywords:** antibodies, IgG polymorphism, Fc gamma receptor, antibody dependent cellular cytotoxicity, glycosylation

## Abstract

Antibody dependent cellular cytotoxicity (ADCC) is an Fc-dependent effector function of IgG important for anti-viral immunity and anti-tumor therapies. NK-cell mediated ADCC is mainly triggered by IgG-subclasses IgG1 and IgG3 through the IgG-Fc-receptor (FcγR) IIIa. Polymorphisms in the immunoglobulin gamma heavy chain gene likely form a layer of variation in the strength of the ADCC-response, but this has never been studied in detail. We produced all 27 known IgG allotypes and assessed FcγRIIIa binding and ADCC activity. While all IgG1, IgG2, and IgG4 allotypes behaved similarly within subclass, large allotype-specific variation was found for IgG3. ADCC capacity was affected by residues 291, 292, and 296 in the CH2 domain through altered affinity or avidity for FcγRIIIa. Furthermore, allotypic variation in hinge length affected ADCC, likely through altered proximity at the immunological synapse. Thus, these functional differences between IgG allotypes have important implications for therapeutic applications and susceptibility to infectious-, allo- or auto-immune diseases.

## Introduction

In the defense against invading pathogens, Immunoglobulins are formed by B cells. These bind the pathogen via their Fab domains and subsequently activate both complement but also immune cells by immunoglobulin Fc-receptors (1). The largest portion are Immunoglobulin γ (IgG) antibodies that mediate their effector functions through Fc gamma receptors (FcγR) on myeloid and Natural Killer (NK) cells (2–4). Antibody dependent cellular cytotoxicity (ADCC) is one of the major Fc-dependent effector functions of IgG, that is particularly important in the clearance of viral infections and is mostly mediated by NK cells (4–6). NK cells mediate ADCC through binding of antibody opsonized target cells by membrane expressed FcγRIIIa and induce cytotoxicity by releasing granzymes and perforins stored in intracellular granules (7, 8). This is an important effector mechanism that contributes to the killing of tumor cells upon immunotherapy (9, 10).

The four IgG subclasses (IgG1, IgG2, IgG3, and IgG4) are defined by unique structural and functional characteristics. Besides variation in terms of half-life, antigen binding and Fab-arm exchange, the subclasses differ in their capacity to activate the complement system or bind FcγR on immune effector cells (2, 11). This variation in responses allows for the generation of tailored antigen-specific responses with optimal effector functions (2, 12–15). The strength of ADCC activity is not only influenced by the IgG subclass, but also by N297-linked glycosylation of the antibody, and FcγR polymorphisms (16–20).

IgG-heavy chain polymorphisms, IgG allotypes, form another layer of variation. Although it is likely that these polymorphisms influence functional and structural features of the IgG subclasses, this has never been systematically studied (2). IgG polymorphic variants were first detected with serological tests, classically by hemagglutination inhibition (21–23). The identification of IgG polymorphisms with serological tests was a powerful tool to study population variations (24). Later, sequencing of the immunoglobulin genes of different ethnic groups revealed that even more polymorphic variants are present that could not be detected with serological testing, in particular for IgG3 (2, 22, 25). IgG polymorphisms were found to be associated with infectious-and auto-immune diseases in multiple studies (26–32), suggesting that IgG allotypes may affect the humoral antibody response. However, the structural or functional characteristics of IgG allotypes underlying these associations remain to be elucidated.

The fact that serological tests are used to determine IgG allotypes, indicates that the amino acid variation in the antibody constant domain is an immunogenic determinant and that therapeutic antibodies potentially induce an anti-allotype response, in the case of an allotype mismatch (33). However, patients treated with IgG1 allotype mismatched anti-TNF therapeutic antibodies were not found to raise anti-allotype antibodies, suggesting that allotypic variation is only a minor immunogenic determinant (34, 35). IgG3 allotypic variants might be more immunogenic, as more amino acid variation is present between allotypes.

IgG allotypes were found to correlate with plasma IgG level, most likely as a result of variation in the non-coding switch regions or unfavorable RNA transcripts. In addition, IgG polymorphisms may also be associated with class-switching efficiency and thereby serum concentrations, through variations within the non-coding switch regions inherited haplotype (36, 37). Recently IgG1 allotypic variants were found to be associated with the subclass distribution (IgG1/IgG2) of an HIV-specific antibody response, illustrating the association of IgG polymorphisms with IgG class switching (38).

In addition to the association of IgG allotypes with antibody expression and IgG class switching in B-cells, the Fc-mediated effector functions are also likely to be different between IgG allotypes. Previous studies already identified IgG3 allotypes with enhanced antibody half-life, IgG3 allotypes with less stable CH3-CH3 interactions and an IgG4 allotype lacking the capacity to exchange half-molecules (13, 14, 39, 40). A particular IgG3 isoallotypic determinant (present in IMGT: IGHG3^∗^17, ^∗^18, and ^∗^19) expressing a histidine at position 435 in the CH3 domain was found to improve pH-dependent binding to the neonatal Fc receptor (FcRn) and therefore showed an half-life that resembled that of IgG1 antibodies (13). The infants of mothers carrying this IgG3 polymorphic variant were found to have an increased protection against malaria, most likely caused by the malaria specific IgG3 antibodies crossing the placental membrane more efficiently as a result of increased binding to FcRn (41, 42).

To capture the structural and functional diversity between IgG allotypes, we generated the complete set of IgG allotypes that have been described to date and compared their binding to FcγR and capacity to induce ADCC *in vitro*. Whereas allotypic variants within the IgG1, IgG2 and IgG4 subclass did not change binding to FcγR or ADCC, the differences in hinge length and CH2 domain between IgG3 allotypes affected FcγRIIIa binding and ADCC substantially. Understanding the functional diversity within IgG subclasses may shed light on associations found with infectious diseases or auto-immune diseases and potentially initiate new strategies to improve therapeutic antibodies.

## Materials and Methods

### Cell Lines

FreeStyle Expi293F cells were cultured in FreeStyle 293 expression medium according to the manufacturer’s instructions (Invitrogen). Additional cell lines were obtained from the American Type Culture Collection (ATCC). Raji (human CD20-positive Burkitt’s lymphoma) cells were cultured in RPMI 1640 medium (Lonza), supplemented with 10% heat-inactivated Donor Bovine Serum with Iron (DBSI; Life Technologies), 4 mM L-glutamine, 25 mM Hepes and 1 mM Sodium Pyruvate (Lonza). Wien-133 (human CD52-positive Burkitt’s lymphoma) cells were cultured in Iscove’s Modified Dulbeco’s Medium (IMDM) with HEPES and L-Glutamine (Lonza), supplemented with 10% heat-inactivated DBSI. All cell lines were maintained at 37°C in a 5% CO_2_ humidified incubator.

### Cloning and Production of Anti-RhD and Anti-TNP Antibodies

All IgG polymorphic variants known at the initiation of the study, deposited in the IMGT-web site, and described previously by Vidarsson et al. (2) were cloned into a pEE6.4 expression vector containing an anti-Rhesus D (anti-RhD) heavy chain of a previously described clone (43). Restriction sites flanking all individual IgG constant domains (CH1, hinge, CH2 and CH3) were introduced to allow the exchange of allotype specific gene fragments (Integrated DNA technologies) and generate all the different polymorphic variants. From the anti-RhD allotype constructs, all 27 IgG allotype constant domains were also cloned in a pcDNA3.1 vector containing the anti-TNP heavy chain by swapping all polymorphic variant constant domains with restriction enzymes *Nhe*I-*Eco*RI (Thermo fisher) (44). Antibodies were expressed by transient transfection of heavy (anti-RhD/anti-TNP) and light chain (anti-RhD/anti-TNP) containing vectors in HEK 293F suspension cells with PEI-MAX (Polyethylenimine Hydrochloride, Linear (MW 4,000), PolySciences), using the HEK 293F expression system, according to the instructions of the manufacturer (Thermo Fisher). Antibodies were purified from the culture supernatant 5 days after transfection using an HiTrap protein A or G column (GE Healthcare). IgG3 allotypes were purified using a protein G column, as protein A does not bind IgG3. After purification, antibodies were concentrated and dialyzed in nanogam buffer (5 mM sodiumacetate + 5% D-Glucose, pH 4.5) and stored in small aliquots at −20°C. Purified IgG allotypes were analyzed for monomeric, dimeric and oligomeric IgG on a Superdex 200 10/300 gel filtration column (30 cm, 24 ml, 17-15175-01, GE Healthcare, Little Chalfont, United Kingdom) connected to an Äkta explorer (GE Healthcare) HPLC system at room temperature with a flow rate of 0.5 ml/min and PBS as running buffer. Elution profiles were obtained by measuring the absorbance at 215 nm.

### Cloning and Production of Anti-CD20 and Anti-CD52 Antibodies

Antibody heavy-chain expression vectors were constructed by inserting *de novo* synthesized (Geneart) codon optimized HC coding regions into expression vector pcDNA3.3 (Invitrogen). The HC coding regions consisted of the VH regions of human mAbs 7D8 [human CD20-specific (45)], Campath [human CD52-specific (46)] or b12 [HIV-1 gp120-specific (47)] genetically fused to the CH regions of human IgG1^∗^03, selected IgG3 allotypes (2), or one of the mutant variants {P291L, R292W, W292R, rch3 [reduced core-hinge consisting of 3 exons], rch1A, rch1B, h1 (G1 hinge), C219S} (EU numbering conventions are used throughout the manuscript). Likewise, separate light-chain expression vectors were constructed by inserting the appropriate VL coding regions in frame with the CL coding regions of the human (J00241) kappa light chain into expression vector pcDNA3.3. All antibodies were produced under serum-free conditions by co-transfecting relevant heavy and light chain expression vectors in FreeStyle Expi293F cells, using ExpiFectamine 293 (LifeTechnologies), according to the manufacturer’s instructions. IgG1 antibodies were purified by protein A affinity chromatography (MabSelect SuRe; GE Health Care), dialyzed overnight to PBS and filter-sterilized over 0.2 μM dead-end filters. Alternatively, IgG3 antibodies were purified by protein G affinity chromatography (GE Health Care). Purity was determined by CE-SDS and concentration was measured by absorbance at 280 nm (specific extinction coefficients were calculated for each protein). Batches of purified antibody were tested by high-performance size-exclusion chromatography (HP-SEC) for aggregates or degradation products and shown to be at least 95% monomeric. Purified antibodies were stored at 2–8°C.

### Liquid Chromatography – Mass Spectrometry Analysis of Immunoglobulin G Glycosylation

Bottom-up glycoproteomics analysis was performed similarly to earlier reports (48). Key aspects and deviations from this protocol are briefly listed in the following. 10 μg IgG were prediluted in 100 μL phosphate-buffered saline (PBS) and added to 2 μL CaptureSelect FcXL beads (agarose beads with immobilized anti-IgG antibody; ThermoFisher Scientific). After 1 h incubation and washing, samples were eluted in 100 μL 100 mM formic acid (analytical grade; Sigma-Aldrich, Steinheim, Germany). Dried samples were re-dissolved in 20 μL 50 mM ammonium bicarbonate to which 0.5 μg TCPK-treated trypsin (Sigma-Aldrich) in 20 μL water were added. Tryptic glycopeptides yielded after overnight incubation were analyzed by RP-nanoLC-MS. 1 μL of a 50-fold diluted sample was injected onto an Acclaim PepMap 100 C18 column 150 × 0.075 mm with 3 μm particles at 700 nL/min flow. The instrumental setup consistent of an Ultimate 3000 RSLC nano LC system (ThermoFisher Scientific) and a maXis quadrupole-time-of-flight-MS (q-TOF) equipped with a nanoBooster nanoESI source (Bruker, Leiden, Netherlands). Ionization parameters were as previously reported (48). A binary gradient of water and 95% acetonitrile (LC-MS grade Biosolve, Valkenswaard, Netherlands) with 0.1% formic acid each consisted of the following steps: 0–5 min 1% B, linear gradient to 27% B 5–20 min, washing at 70% B 21–23 min, and re-equilibration at 1% B 24–42 min. LC-MS data was automatically (pre-)processed with LaCyTools version 1.1.0 alpha build 190207a as previously described, albeit with an extraction window of 65 mTh and without the need to align or calibrate (48, 49).

### Human FcγR Constructs and Control Antibodies

Human FcγR constructs FcγRIa (his tag, 10256-H08H-100), FcγRIIa (131His, biotinylated, 10374-H27H1-B-50 and 131Arg, biotinylated, 10374-H27H-B-50), FcγRIIb (biotinylated, 10259-H27H-B-50), and FcγRIIIa (158Phe, biotinylated, 10389-H27H-B-50, and 158Val, biotinylated, 10389-H27H1-B-50) for surface plasmon resonance (SPR) analysis were obtained from Sino Biological (Beijing, China). We used Fc-FcγRIIIB fusion proteins to determine binding affinities to two polymorphic variants of FcγRIIIB (NA1 and NA2), as described previously (50).

### Surface Plasmon Resonance (SPR)

Affinity measurements were essentially performed with the IBIS MX96 biosensor system as described previously (18, 20). In short, all biotinylated human FcγR were spotted using a continuous flow micro spotter (Wasatch Microfluidics, Salt Lake City, UT, United States) onto a SensEye G-Streptavidin sensor (Senss, Enschede, Netherlands) at four different densities 1, 3, 10, and 30 nM. A 2-fold dilution series of IgG allotypes (0.49 to 1000 nM) were flowed over the chip starting with the lowest antibody concentration. For FcγRIa affinity measurement, a biotinylated anti-His antibody (Genscript) was spotted at four different densities (1, 3, 10, and 30 nM) and prior to each antibody injection 50 nM his-tagged FcγRIa was flowed over the sensor. Regeneration after each sample was carried out with 10 nM Glycine HCL pH 2,4. K_*d*_ values were obtained by equilibrium fitting (Scrubber) and interpolating to Rmax 500 (Excel).

### Cellular Surface Plasmon Resonance (cSPR)

Rhesus D positive (RhD+) red blood cells (RBC) were obtained from the serology department (donor 18-955, R1R1 genotype) and opsonized with different IgG3 allotypes in a concentration range from 2.5 μg/ml in a 1:2 dilution to 0.078 μg/ml. We confirmed by eye that the RBC did not form aggregates after opsonization. After four wash steps with PBS to remove unbound antibody, the opsonized RBCs were resuspended in 300 μl PBS/0.1% BSA at a concentration of 0.5 × 10^8^ RBCs/ml. Biotinylated FcγR were spotted using a continuous flow micro spotter (Wasatch Microfluidics, Salt Lake City, UT, United States) onto a SensEye G-Streptavidin sensor (Senss, Enschede, Netherlands) at two different densities (10 and 30 nM) in quadruplo (four spots per condition). To control for opsonization levels between allotypes, biotin anti-LC-kappa (Thermo Fisher) was spotted at 1 nM and 5 nM.

The opsonized RBCs were injected over the sensor, after which the flow was stopped to allow sedimentation of the RBCs on the sensor for 5 min (sedimentation phase/S). Specific binding was detected by step-wise increasing the flow speed (1, 2, 4, 8, 10, 20, 40, 80, and 120 μl/s), where unbound cells are flushed away and bound cells are interacting stronger with the chip. Regeneration after each sample was performed with 10 nM Glycine HCL pH 2,8 containing 0.075% Tween-80. Specific binding was measured in response units (RU) after the end of each flow cycle (T). This signal was divided by the RU after the sedimentation phase (S) and expressed as T/S ratios.

### Antibody Dependent Cellular Cytotoxicity (ADCC) With Anti-CD20 and Anti-CD52 Antibodies

Peripheral blood mononuclear cells (PBMC) were isolated from blood by density gradient centrifugation using Leucosep tubes (Greiner Bio-one), according to the manufacturer’s instructions. Briefly, buffy coats from standard blood donations (Sanquin Blood Bank) were diluted 3.6-fold in PBS and layered on top of 15 mL Lymphocyte Seperation Medium in 50 ml tubes. Tubes were subsequently centrifuged at 800 × *g* for 15 min at 20°C and PBMCs were recovered from the plasma-medium interface. Collected PBMCs were then washed 3 times with 50 mL of PBS followed by centrifugation for 10 min at 300 × *g*. Isolated PBMCs were resuspended in culture medium (RPMI-1640 medium containing 2 mM L-glutamine and 25 mM Hepes (Lonza) supplemented with 10% donor bovine serum with iron [DBSI, Life Technologies)].

To determine the ADCC capacity of anti-CD20 and anti-CD52 antibodies, the DELFIA EuTDA TRF (time-resolved fluorescence) cytotoxicity kit (Perkin Elmer) was used, according to manufacturer’s instructions. In short, Raji or Wien-133 target cells (T) were resuspended at a concentration of 1 × 10^6^ cells/mL in culture medium (see above) and labeled with 0.16% (v/v) or 0.33% (v/v) bis(acetoxymethyl)2,2′:6′,2″-terpyridine-6,6″-dicarboxylate reagent solution (DELFIA BATDA reagent, Perkin Elmer), respectively, for 20 min at 37°C in a water bath. The hydrophobic BATDA label is intracellularly converted into the hydrophilic TDA label (2,2′:6′,2″-terpyridine-6,6″-dicarboxylic acid) which is than retained intracellularly. Labeled cells were subsequently washed three times with culture medium to remove excess BATDA label. Labeled cells were mixed with 4-fold serial dilutions of various anti-CD20 and anti-CD52 antibodies and pre-incubated for 15 min at 20°C. Next, freshly isolated PBMC effector cells (E) were added to the mixture at a E:T ratio of 100:1 in culture medium in a total volume of 200 μL in a V-bottom 96-well plate. The actual E:T ratio is estimated at 5:1 since only CD16 expressing cells (NK-cells and NKT cells) in the PBMC population are considered to participate in ADCC. Plates were incubated for 2 h at 37°C in a 5% CO_2_ humidified incubator. Spontaneous TDA release from labeled cells was determined in the absence of PBMCs and antibodies and maximum TDA release was determined by incubating labeled cells with 0.1% (v/v) Triton X-100 (Sigma Aldrich). After 2 h plates centrifuged for 5 min at 500 × *g* and 20 μL of cell-free supernatant was transferred into a flat bottom 96-well white opaque OptiPlate (Perkin Elmer). Subsequently, 200 μL of DELFIA Europium Solution (Perkin Elmer) was added to the transferred supernatant and samples were incubated for 15 min at 20°C in the dark. The fluorescence of the EuTDA chelates formed from the released TDA label was measured in a Wallac 2104 EnVision multilabel plate reader (Perkin Elmer) with a UV 340 excitation filter and a Europium 615 emission filter. Percentage lysis was calculated using the following formula:% lysis = [experimental release (fluorescence) – spontaneous release (fluorescence)]/[maximal release (fluorescence) – spontaneous release (fluorescence)] × 100. Specific lysis was calculated by subtracting the background lysis (resulting from target and effector cells in the absence of antibody). Per donor each condition was tested in triplicate.

### Antibody Dependent Cellular Cytotoxicity (ADCC) With Anti-RhD and Anti-TNP Antibodies

Whole blood was drawn from healthy volunteers and collected in heparin containing tubes to prevent clotting. NK cells were isolated from Ficoll-Plaque-Plus (GE Healthcare) gradient obtained PBMCs by a CD56 magnetic activated cell separation (MACS) isolation kit (Miltenyi Biotec, Leiden, Netherlands), according to manufacturer’s description. After isolation, cells were incubated over night in Iscove’s modified dulbecco’s medium (IMDM, Gibco, Thermo Fisher Scientific) supplemented with 10% fetal calf serum (FCS, Bodinco, Alkmaar, Netherlands) to allow dissociation of all cytophilic antibodies bound to the CD16 (FcγRIIIa) receptors. To determine the ADCC capacity of the anti-RhD allotypes, bromelain treated RhD+ red blood cells (donor 18-1000, R2R2 genotype, provided by erythrocyte serology department) were used. Bromelain treatment removes the glycocalyx barrier of RBCs and thereby enhances the ADCC capacity of fucosylated anti-RhD antibodies (16). To study the ADCC capacity of anti-TNP IgG allotypes, untreated RBC (donor 18-1000) were incubated with a 0.25 mM 2,4,6-trinitrobenzenesulfonic acid (TNBS) solution (Sigma-Aldrich) for 10 min at room temperature and then washed twice with PBS to remove unbound TNP.

In general, ADCC experiments were performed as described previously (18). Briefly, RBCs were labeled with radioactive chromium (100 μCi ^51^Cr, PerkinElmer, Waltham, MA, United States) at 10^9^ cells/ml for 45 minutes at 37°C and then washed twice with PBS to remove unbound ^51^Cr. 10^5^ RBCs were mixed with NK cells at a ratio of 2:1 in IMDM medium supplemented with 10% FCS in a V-bottom 96-wells plate. The various IgG allotypes were tested in a total volume of 100 μl at a final concentration of 1.25 μg/ml, unless stated otherwise. Plates were spun down for 1 min at 330 × *g* and subsequently incubated for 2h at 37°C. Spontaneous ^51^Cr release from RBCs (background) was determined in the absence of NK-cells and IgG and maximum ^51^Cr release was determined by incubating RBCs with 2.5% saponin. After 2 h supernatants were collected and released ^51^Cr was quantified in a Packard Cobra II Auto-Gamma Counter Model D5005 (PerkinElmer).

Percentage cytotoxicity was determined by the following formula (cpm = counts per minute):

Killing%=[(cpm-samplecpm)background/(cpm-maximalcpm)background]⋅100

Each condition was at least tested in three individual sample wells. To combine ADCC data of different NK cell donors, relative ADCC capacity was quantified by normalizing the data to IgG3 allotype ^∗^01. Thus, for each individual experiment the % ADCC obtained with a certain IgG allotype was divided by the % ADCC obtained with IgG3 allotype ^∗^01 in the same experiment.

### Statistics

Statistical analysis was performed with GraphPad Prism software version 8 (GraphPad Software, United States). Significant differences were determined by One-way ANOVA with Sidek’s multiple comparisons test.

### Ethics Statement

All donors were informed on the donor privacy regulation of their blood donation and provided informed consent with their approval for research purposes.

## Results

### Production of IgG Allotypes With Anti-RhD and Anti-TNP Specificity

**FIGURE 1:**
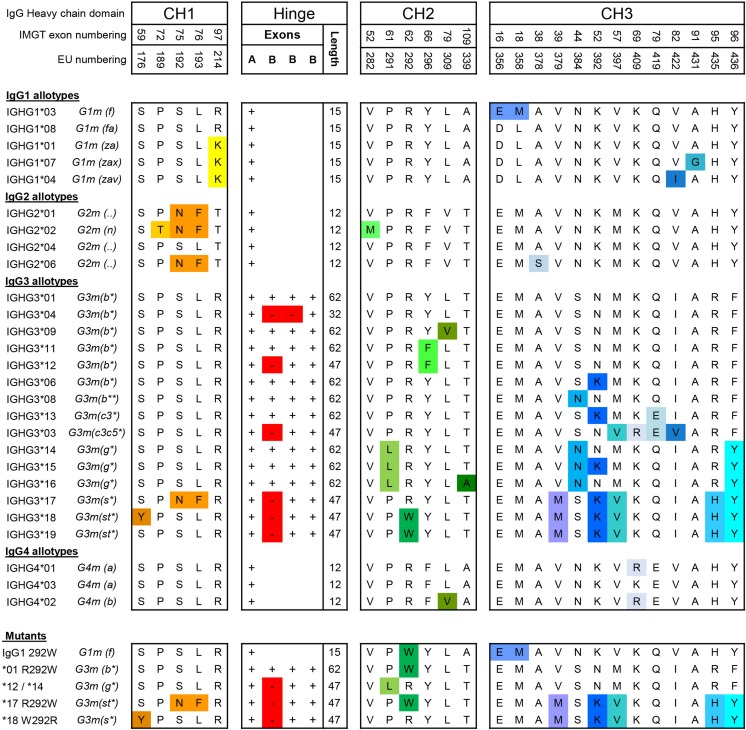
Amino acid variation between IgG allotypes. Variation at the amino acid level between IgG allotypes within the IgG1, IgG2, IgG3, and IgG4 subclasses and mutants [Adapted from Vidarsson et al. 2014 (2)]. For each domain, CH1, hinge, CH2 and CH3, amino acid differences between polymorphic variants are indicated with specific colors. Polymorphisms in the hinge region are identified by the presence or absence of hinge exons (A and B).

**FIGURE 2:**
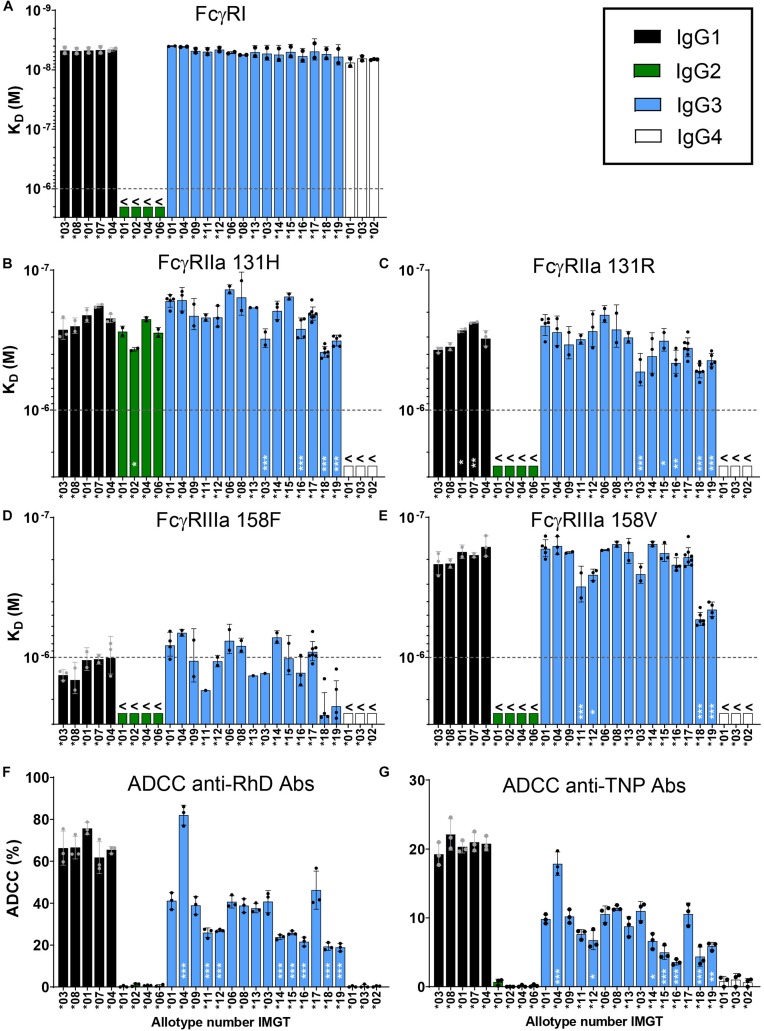
Characterization of binding strength of IgG allotypes to FcγR. Binding of immunoglobulin G allotypes to **(A)** FcγRI, **(B)** FcγRIIa 131H, **(C)** FcγRIIa 131R, **(D)** FcγRIIIa 158F and **(E)** FcγRIIIa 158V as determined by SPR. K_*D*_ values for each IgG allotype are plotted as bar graphs, in which black bars represent IgG1 allotypes, green bars IgG2 allotypes, blue bars IgG3 allotypes and white bars IgG4 allotypes. The highest antibody concentration (1000 nM/1 × 10^– 6^ M) that was used in the SPR measurement is depicted as a dotted line. K_*D*_ values below this line are unreliable and are represented as >1000 nM. Error bars indicate SEM of ≥2 independent measurements. Individual sensorgrams from which K_*D*_ values were quantified are displayed in Supplementary Figure S1B. NK cell mediated killing of **(F)** bromelain treated RhD+ red blood cells by all anti-RhD allotypes and **(G)** TNPlated red blood cells by all anti-TNP allotypes at a concentration of 1.25 μg/ml. Percentage ADCC specific killing of red blood cells was measured in triplo and the mean is plotted in a bar graph. NK-cell mediated ADCC by anti-RhD allotypes was measured with NK cells from four individual donors, as shown in Supplementary Figure S2A. One representative result of four individual experiments is depicted here. To determine significant differences between allotypes within each subclass we used a one-way ANOVA with Sidak’s multiple comparison test, and significant differences are indicated with white asterisks: *p < 0.05, **p < 0.01, ***p < 0.001. This statistical analysis was performed separately for all allotypes within a subclass and compared to one reference antibody (IgG1*03, IgG2*01, IgG3*01, and IgG4*01).

To study the influence of IgG polymorphisms on the ADCC activity, all polymorphic variant constant domains described previously were paired with variable domains with anti-RhD specificity or anti-TNP specificity and expressed in HEK293F cells with the corresponding light chain (42–44). We refer to unique polymorphic variants by using the IMGT allele names, instead of the numerical or alphabetical allotype nomenclature as we described previously (Figures 1, 2). To confirm the production of monomeric IgG, we analyzed the purified anti-RhD IgG allotypes by HPLC-SEC. All antibodies eluted from the column as a single monomeric fraction, without the presence of aggregates (Supplementary Figure S1A).

### IgG Allotypes Display Small Differences in Affinity for Fc-Gamma Receptors

Next we used the IBIS MX96 biosensor system to determine the binding strength of the IgG allotypes (anti-RhD specificity) to all activating human FcγR, which are involved in Fc mediated effector functions of IgG such as ADCC (18). This was an exploratory binding study to identify allotypic determinants influencing FcγR binding. In general, binding patterns for the IgG subclasses to FcγR Ia, IIa (131H and 131R), IIb, IIIa (158F and 158V), IIIb (NA1 and NA2) were in line with data reported previously (Figures 2A–E and Supplementary Figure S1B) (20, 51, 52). The interaction of IgG allotypes with FcγRIIb and FcγRIIIb (NA1 and NA2) was too weak to calculate binding affinities (>1000 nM; Supplementary Figure S1B). We found that the affinity of IgG3 (allotype ^∗^01) to FcγR is slightly higher compared to IgG1 (allotype ^∗^03) and confirmed this across three different specificities (Supplementary Figure S1C). These subtle differences in affinity to FcγR between IgG1 and IgG3 are not always consistent with previously published studies, which likely originates from differences in SPR set-up and is dependent on the glycosylation and allotype of the antibody used in the analysis (20, 51, 52). Within the IgG1 subclass we did not observe differences between the allotypes in affinity for FcγR, except for a small increase in binding to FcγRIIa 131R for allotypes ^∗^01 and ^∗^07 compared to ^∗^03, indicating that the allotypic variations within IgG1 are not influencing binding to FcγR to a substantial degree (Figures 2B–E). Within the IgG2 and IgG4 subclasses, the allotypic variants also showed similar binding to the FcγR. However, when comparing IgG3 allotypes, relatively small (≤3-fold) but consistent differences were observed in binding affinities for both FcγRIIa and FcγRIIIa (Figures 2B–E). Most prominently, compared to other IgG3 allotypes, IGHG3^∗^18 and ^∗^19 bound FcγRIIIa (158V and 158F polymorphic variant) with a 3-fold reduced affinity (*p* < 0.001; Figures 2D,E). A unique feature that the allotypes IGHG3^∗^18 and ^∗^19 have in common is that they display a tryptophan at position 292 in the CH2 domain instead of an arginine, which might affect binding to FcγRIIIa (Figure 1). Consistent with previous studies, the affinity of IgG1 and IgG3 allotypes for FcγRIIIa was highly dependent on the polymorphic variant of FcγRIIIa with the V158 variant binding with significantly higher affinity (18, 20, 51) (Figures 2D,E).

### The Potency to Induce ADCC Is Different Between IgG3 Allotypes

Next, we compared the potency of all anti-RhD allotypes in an *in vitro* ADCC assay. The IgG2 and IgG4 allotypes did not induce NK cell mediated ADCC activity, which was in line with the very weak binding to FcγRIIIa (Figure 2F and Supplementary Figure S2A). Within the IgG1 subclass, all allotypes efficiently and similarly induced ADCC (∼70% killing) and no differences were observed across IgG1 allotypes (Figure 2F). Amongst the IgG3 allotypes large variation was observed in the capacity to induce ADCC, ranging from 20 to 80% killing of target cells, which only partially matches the affinity differences measured by SPR (Figures 2D–F). To determine whether our findings were antigen-dependent, we also analyzed the ADCC capacity of anti-TNP IgG allotypes in a parallel experiment in which TNP-lated RBC were used as target cells (Figure 2G). Overall, the ADCC data with anti-TNP allotypes is remarkably similar to that observed with the anti-RhD antibodies (*r* = 0.88, *p* > 0.001, Supplementary Figure S2C), suggesting that the effect of the IgG3 allotypic determinants on ADCC activity is antigen-independent (Figures 2F,G).

### The Length of the Hinge Influences the Capacity of IgG3 Allotypes to Induce ADCC

**FIGURE 3:**
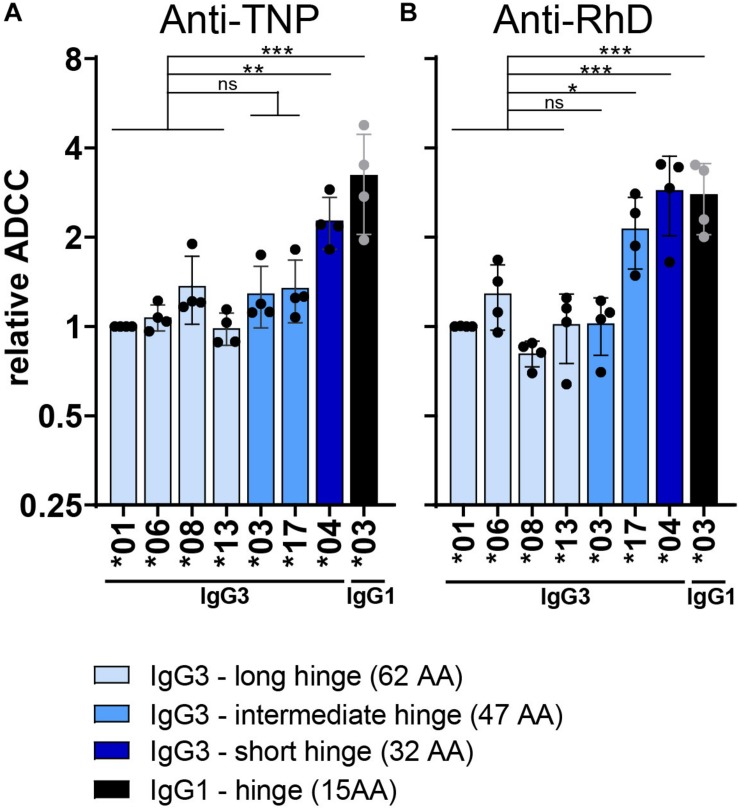
Influence of the hinge on ADCC capacity of IgG3 allotypes. Relative ADCC activity of both **(A)** anti-TNP and **(B)** anti-RhD antibodies with varying hinge length. ADCC activity of all IgG allotypes was performed with NK-cells isolated from four different donors and is shown in Supplementary Figures S2, S4. Depicted here is the relative ADCC compared to IgG3*01 (set to 1) derived from the raw ADCC data. Only IgG3 allotypes with a PRYLT CH2 domain (Figure 1) are plotted to exclusively monitor the effect of the hinge length. IgG3 allotypes are colored according to their hinge length, with long hinge allotypes in light blue, intermediate hinge in blue and short hinge allotypes in dark blue. Black bars depict the relative ADCC capacity of IgG1 allotype *03 as a comparison. Statistical comparison between groups was performed using a one-way ANOVA with Sidak’s multiple comparison test, and significant differences are indicated with asterisks: *p < 0.05, **p < 0.01, ***p < 0.001. The broad line above the long hinge variants indicates that the statistical comparison between for example allotype *04 and the four long hinge variants (all tested individually) had the same outcome.

To identify structural determinants influencing IgG3-mediated ADCC, the role of the hinge length was examined more closely. IgG3 allotypes that express only 3 hinge exons (^∗^03 and ^∗^17) show a trend toward higher ADCC capacity compared to the long hinge allotypes (4 hinge exons) (Figure 3). ADCC activity of IgG3^∗^17 was antigen or context dependent, only enhancing ADCC activity compared to allotype ^∗^01 (*p* = 0.02) in the anti-RhD background. This context dependency of IgG3^∗^17 was also observed in a recent study analyzing ADCC activity of two anti-HIV-1 antibodies (52). The IgG3 allotype with a short hinge, IGHG3^∗^04 (2 hinge exons), showed a 2-fold enhanced (*p* > 0.001 in anti-RhD and *p* = 0.01 in anti-TNP background) ADCC capacity compared to allotypes with a long hinge (Supplementary Figure S2A and Figures 3A,B). The hinge of IgG1 antibodies is even shorter than that of the short hinge IgG3 allotype IGHG3^∗^04 and this characteristic appears to explain the strong ADCC capacity of IgG1 compared to most IgG3 allotypes (3-fold increase compared to longe hinge IgG3 allotypes: *p* > 0.001 in anti-RhD and *p* > 0.001 in anti-TNP background) (Figures 3A,B). These findings were corroborated using a matched set of anti-CD20 IgG3 hinge-length variants in an alternative (PBMC-based) ADCC assay investigating Raji cell lysis (Supplementary Figure S3). In conclusion, IgG3 allotypes with a short hinge show stronger induction of ADCC compared to allotypes with a long hinge, while having a similar affinity for FcγRIIIa.

### Residues in the IgG3-CH2 Domain Affect ADCC

Several variants showed a consistent deviation in ADCC activity (Figures 2F,G) that was not explained by altered hinge length, but rather seemed to stem from unique composition in the CH2-region. To analyze this in more detail we plotted the ADCC data for all allotypes that have a similar hinge length (4 exons), but have a different CH2 composition (Supplementary Figure S2B). Allotype ^∗^09, bearing a unique valine at position 309 (Figure 1), showed no altered ADCC, in agreement with no effect on affinity to FcγRIIIa (Supplementary Figure S2B and Figures 2D,E). Conversely, allotypes with a phenylalanine at position 296 (^∗^11 and ^∗^12), or a tryptophan at position 292 (^∗^18 and ^∗^19) showed both lowered affinity and ADCC (Supplementary Figure S2B and Figures 2D,E). Remarkably, IgG3 allotypes with a leucine at position 291 (^∗^14, ^∗^15, and ^∗^16) also showed lowered ADCC, but without apparent changes in affinity (Supplementary Figure S2B and Figures 2D,E).

### IgG With a Tryptophan at Position 292 Reduces FcγRIIIa Binding and ADCC Efficiency

**FIGURE 4:**
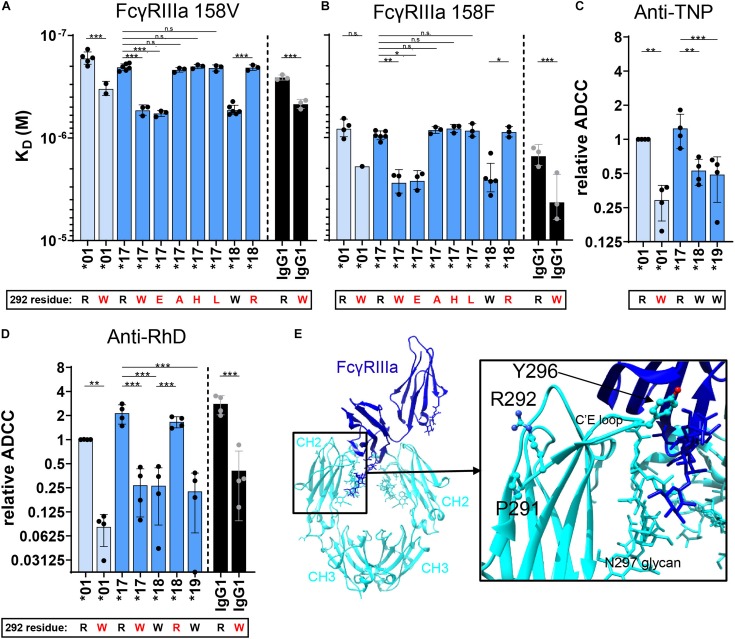
FcγRIIIa binding and ADCC capacity of 292-mutated IgG allotypes. The affinity (K_*D*_) of 292–mutant anti-RhD IgG allotypes for **(A)** FcγRIIIa 158V and **(B)** FcγRIIIa 158F was determined by SPR. Error bars indicate SEM of ≥2 independent measurements. ADCC activity was assessed for 292-mutant **(C)** anti-TNP allotypes and **(D)** anti-RhD antibodies using NK cells from four different donors. The relative ADCC capacity compared to IgG3 allotype *01 is plotted (original ADCC data in Supplementary Figure S4). IgG3 allotypes and 292-mutants with a long hinge are displayed in light blue, an intermediate hinge in blue, a short hinge in dark blue and IgG1 in black. In all graphs the amino acid at residue 292 (single-letter code) for each antibody is indicated at the *x*-axis below the IgG3 allotype number, where 292-mutated antibodies are displayed with a red letter. Statistical comparison between groups was performed using a One-way ANOVA with Sidak’s multiple comparisons test, and significant differences are indicated with asterisks: *p < 0.05, **p < 0.01, ***p < 0.001. **(E)** Crystal structure of fucosylated IgG1 Fc (cyan) in complex with FcγRIIIa (blue) (PDB; 3SGJ) (53). Residues Pro-291, Arg-292 and Tyr-296 are displayed in a ball-and-stick model.

We further investigated whether the tryptophan at residue 292 in the CH2 domain of IgG3 allotype ^∗^18 and ^∗^19 is responsible for reduced FcγRIIIa binding and ADCC capacity. The R292W mutation significantly reduced the affinity of IGHG3^∗^17 for FcγRIIIa (158V and 158F) and resulted in an affinity that resembled that of IGHG3^∗^18 (natural W292 allotype) (Figures 4A,B). A similar reduction in affinity for FcγRIIIa was observed when the R292W mutation was introduced in IgG1 (IGHG1^∗^03) or another IgG3 allotype (IGHG3^∗^01) (Figures 4A,B). Substituting the W292 for an R292 (W292R) in IGHG3^∗^18 improved the affinity for FcγRIIIa to an affinity that resembled that of IGHG3^∗^17. The affinity for both FcγRIIIa 158V and 158F was affected by the 292 mutants, indicating that the effect is not FcγRIIIa polymorphism-specific (Figures 4A,B). These binding studies provide evidence that the presence of a W292 in IgG3 allotype ^∗^18 and ^∗^19 decreases binding to FcγRIIIa and hence ADCC.

The crystal structure of IgG1-Fc in complex with FcγRIIIa implies that residue 292 indirectly affects binding to FcγRIIIa, since it is not directly positioned in the binding interface of IgG-Fc with FcγRIIIa (Figure 4E) (53). To determine whether the effect on FcγRIIIa binding is tryptophan-specific, we introduced other amino acids, either small (R292A), negatively charged (R292E), hydrophobic (R292L), or positively charged (R292H) in IgG3 allotype ^∗^17. Interestingly, only the introduction of a negatively charged residue (R292E) reduced affinity for FcγRIIIa to a similar extent as the introduction of a tryptophan, which suggests that the introduction of an amino acid with largely opposing characteristics compared to arginine also alters the conformation of the CH2 domain in such a way that FcγRIIIa binding is impaired (Figures 4A,B).

Next, we studied the induction of ADCC by selected 292 mutants. We found that the small differences in affinity for FcγRIIIa between R292 and W292 antibodies are directly translated to ADCC efficiency (Figures 4C,D and Supplementary Figures S4, S5A). Furthermore, the W292 anti-RhD mutants showed consistently reduced ADCC activity, independent of the IgG subclass or hinge length (Figure 4D and Supplementary Figure S5A). This finding was confirmed with an IgG3^∗^01 R292W anti-TNP mutant and several W292 anti-CD52 mutants (Figure 4C and Supplementary Figure S5C), although the effects were most profound for anti-RhD. The ADCC activity of the anti-CD20 IgG3 variants was not potent enough to study these differences. However, in the anti-CD20 IgG1^∗^03 context the R292W mutation clearly affected ADCC (Supplementary Figure S5B).

### IgG With a Leucine at Position 291 Reduces ADCC Capacity

**FIGURE 5:**
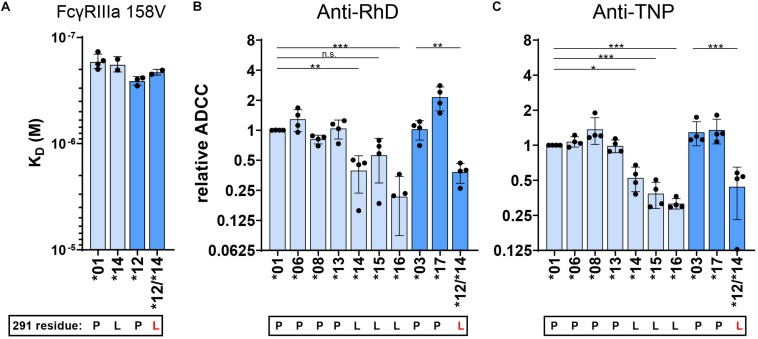
FcγRIIIa binding and ADCC capacity of 291-mutated IgG3 allotypes. **(A)** The affinity of 291–mutant and natural anti-RhD IgG allotypes for FcγRIIIa 158V was determined by SPR. K_*D*_ values of at least two individual measurements are plotted. ADCC activity was determined for 291-mutant **(B)** anti-RhD and **(C)** anti-TNP allotypes using NK cells from four different donors. The relative ADCC capacity compared to IgG3 allotype *01 is plotted (original ADCC data in Supplementary Figure S4). IgG3 allotypes and 291-mutants with a long hinge are displayed in light blue and with an intermediate hinge in blue. In all graphs the amino acid at residue 291 (single-letter code) for each antibody is indicated at the *x*-axis below the IgG3 allotype number, where 291-mutated antibodies are displayed with a red letter. Mutant *12/*14 contains two mutations in the CH2 domain (F296Y and P291L) compared to IgG3 allotype *12. Statistical comparison between antibodies were performed using a one way ANOVA analysis with Sidak’s multiple comparisons test (*p < 0.05, **p < 0.01, ***p < 0.001).

We next explored whether the presence of a leucine instead of a proline at position 291, unique to IgG3 allotypes ^∗^14,^∗^15, and ^∗^16, is responsible for their low ADCC capacity, despite no apparent effect on their FcγRIIIa affinity. We first swapped the CH2 domain of IgG3 allotype ^∗^14 (containing a leucine at position 291) into allotype IgG3 ^∗^12 (mutant ^∗^12/^∗^14, Figure 1). The resulting variant showed no significant changes in affinity compared to the parental antibody (Figure 5A). However, the ADCC capacity of mutant ^∗^12/^∗^14 was decreased to a similar ADCC activity as the natural L291 bearing IgG3 allotypes, indicating that the leucine at position 291 negatively affects the induction of ADCC by IgG3 (Figures 5B,C and Supplementary Figure S4). This was true for both anti-RhD and anti-TNP antibodies, irrespective of the hinge length (Figures 5B,C and Supplementary Figure S4). The effect of the leucine at position 291 was also confirmed for IgG1 in the context of anti-CD20 IgG1^∗^03-mediated ADCC of Raji cells (Supplementary Figure S5D). Curiously, this effect was neither seen for the L291-IgG1 nor for IgG3 variants in the context of anti-CD52 variants (Supplementary Figure S5E), suggesting some context-dependency.

### L291 and W292 Negatively Affect Avidity to FcγR

**FIGURE 6:**
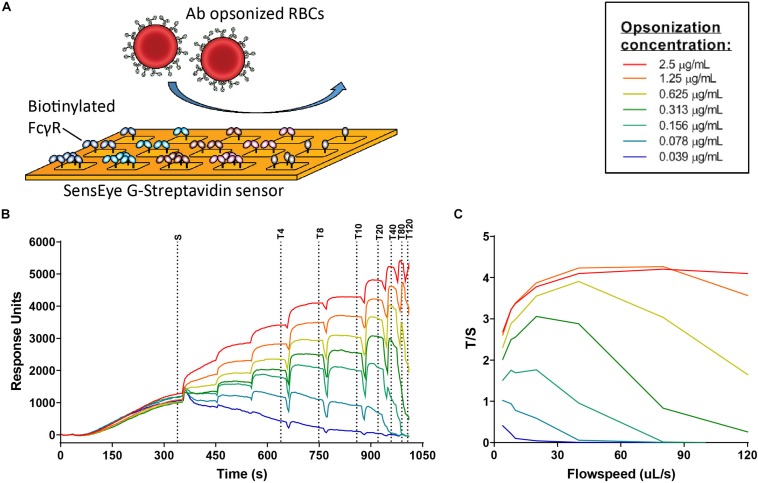
FcγR avidity measurements using cellular SPR. **(A)** Schematic figure of cellular surface plamon resonance (cSPR) avidity measurements showing FcγR spotted on the sensor at different densities and subsequently flow of opsonized red blood cells over the sensor **(B)** Raw sensorgram of cSPR avidity measurement in which the sedimentation phase (S) and increasing flow speeds are indicated. Each color represents a particular level of antibody opsonization, ranging from 2.5 μg/ml to 0.039 μg/ml. **(C)** Total/sedimentation (T/S) response units (RU) ratios are plotted to illustrate binding strength of RBCs opsonized with IgG3 allotype IGHG3*01 to FcγRIIIa 158F at each flow speed.

**FIGURE 7:**
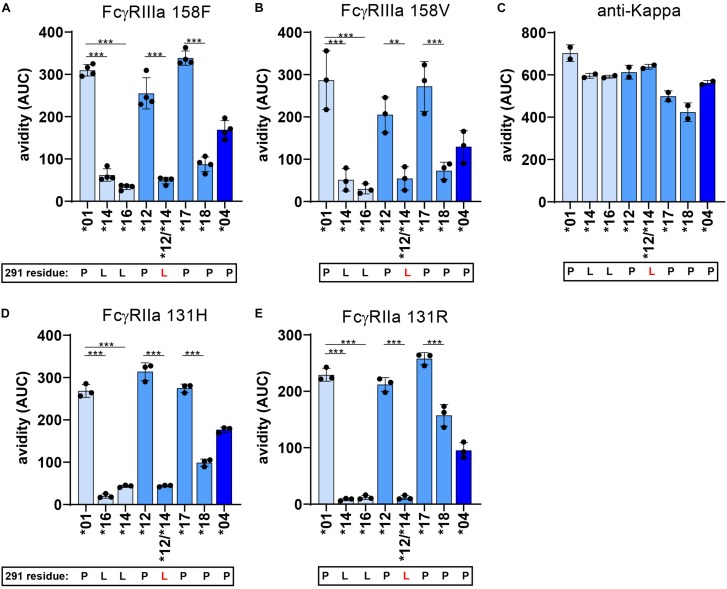
FcγR avidity measurements of IgG3 allotypes. The avidity of RBCs opsonized with various IgG3 allotypes to FcγR as determined by cSPR. Avidity measurements to FcγRIIa 131R, FcγRIIa 131H, FcγRIIIa 158F, FcγRIIIa 158V were determined for seven IgG3 allotypes (*01, *04, *12, *14, *16, *17, *18) and one IgG3 mutant expressing a L291 (*12/*14). To compare between allotypes we calculated area under the curve (AUC) values from the Total/Sedimentation (T/S) ratios that are plotted in Supplementary Figure S6 at a specific receptor density and RBC opsonization concentration. Thus, avidity measurements to **(A)** FcγRIIa 131R at a receptor density of 30 nM and opsonization concentration of 0.625 μg/ml, **(B)** FcγRIIa 131H at a receptor density of 30 nM and opsonization concentration of 2.5 μg/ml, **(C)** FcγRIIIa 158F at a receptor density of 30 nM and opsonization concentration of 0.625 μg/ml and **(D)** FcγRIIIa 158V at a receptor density of 10 nM and opsonization concentration of 1.25 μg/ml. **(E)** Binding strength to an anti-kappa nanobody (density of 1 nM and opsonization concentration of 0.625 μg/ml) was determined simultaneously to confirm equal RBC opsonization levels with each allotype. Error bars indicate SD of ≥2 independent measurements. In all graphs the amino acid at residue 291 (single-letter code) for each antibody is indicated at the *x*-axis below the IgG3 allotype number, where 291-mutated antibodies are displayed with a red letter. Statistical comparison between antibodies were performed using a one way ANOVA analysis with Sidak’s multiple comparisons test (*p < 0.05, **p < 0.01, ***p < 0.001).

To further investigate the strong functional difference between L291- and P291-expression variants despite lack of noticeable affinity differences for FcγRIIIa, we used a cellular SPR setting which measures avidity rather than affinity (Figure 6A) (54–56). Small differences in affinity may not easily be measured directly, but may result in observable larger differences in avidity. We therefore opsonized RBC with the anti-RhD IgG3 antibodies and analyzed binding strength of the opsonized RBC to a FcγRIIIa spotted streptavidin-sensor at different flow speeds (Figures 6A–C). The binding strength of opsonized RBC to a biotinylated anti-kappa light chain nanobody was measured simultaneously and confirmed equal opsonization levels between allotypes (Figure 7 and Supplementary Figure S6). The binding strength of RBC opsonized with IgG3 allotypes bearing the L291 (IGHG3^∗^14 and ^∗^16) to FcγRIIIa (158F and 158V) was lower compared to P291 bearing allotypes (IGHG3^∗^01, ^∗^04, ^∗^12, and ^∗^17), which is in line with the reduced capacity of these allotypes to induce ADCC (Figure 7 and Supplementary Figure S6). A similar reduction in avidity was observed for the RBCs opsonized with IgG3 mutant ^∗^12/^∗^14, which also expresses a leucine at position 291 (Figure 7). Interestingly, binding strength to FcγRIIa (131H and 131R) was also reduced for RBCs opsonized with a L291 expressing anti-RhD antibody (^∗^14, ^∗^16, and ^∗^12/14) (Figure 7). For allotype IGHG3^∗^18 bearing a W292 a reduced affinity for FcγRIIIa was already observed in the conventional SPR. In the cellular SPR assay we were able to confirm the weak binding to FcγRIIIa for RBCs opsonized with a W292 expressing allotype (IGHG3^∗^18) (Figure 7).

### IgG3 Allotypes Have Different Fc-Glycosylation Patterns

The composition of the N297 Fc-glycan has previously been described to influence binding to FcγR and subclass differences in glycosylation are well established (18). We explored whether the differences in FcγR binding and ADCC activity between allotypes are related to the *N*-linked glycosylation profile. In general, glycosylation patterns between allotypes within subclasses were quite similar in terms of fucosylation and sialylation (Supplementary Figures S7A,B). Substantial differences in bisection and galactosylation were observed between IgG3 allotypes, which was consistent for both the anti-RhD and anti-TNP IgG3 allotypes (Supplementary Figures S7A–C). Furthermore, the galactosylation and bisection levels of a 291L and 291W anti-TNP mutant variants were higher compared to their unmutated counterparts, illustrating the influence of these allotypic determinants on *N*-linked glycosylation (Supplementary Figure S7B). It is unlikely that these differences explain the functional differences between allotypes, since galactosylation or bisection level did not or very weakly correlate with FcγRIIIa binding strength or ADCC activity (Supplementary Figures S7D,E).

## Discussion

We studied FcγR binding and ADCC activity of all described IgG polymorphic variants in the human population and identified several determinants in the hinge and CH2 domain of IgG3 allotypes that affect FcγRIIIa binding and ADCC activity. We found that the extensive allotypic variation found in the CH2 domain of IgG3 allotypes translates to functional differences in NK-cell mediated ADCC activities through altered avidity to FcγRIIIa. The amino acid variation between IgG3 allotypes also altered the *N*-linked glycosylation profile. In addition, we found that the natural variation in the IgG3-hinge length also influences NK-cell mediated ADCC activity, suggesting proximity between effector cell and target cell to be a critical factor in determining ADCC potency. In general, the effect of CH2 and hinge determinants on ADCC capacity of IgG allotypes was consistent using various target cells (RBC, Raji, Wien133), expressing different antigens (RhD, TNP, CD20, CD52). The magnitude of the effect was, however, stronger using anti-RhD antibodies compared to a setting where anti-CD52 antibodies and Wien133 cells were used, indicating that the influence of the allotypic determinants is context-dependent. Potential factors that determine these differences include antigen mobility, antigen density, epitope topology and target cell membrane characteristics.

All allotypic variations in the CH2 domain of IgG3 that influence ADCC activity are positioned proximally to the N297 glycan and close to the FcγRIIIa-binding site. This likely explains why the glycosylation profile was affected by these determinants, i.e., high galactosylation and bisection levels for 291L and 292W expressing IgG allotypes and mutants. Although high galactosylation and bisection have been described to increase binding affinity to FcγRIIIa (57–59), our data suggests that the glycosylation profile of the IgG3 allotypes only had a minimal influence on Fc receptor binding and ADCC activity. Residue 296 was previously described to be involved in the direct interaction with FcγRIIIa and mutating Y296 in IgG1 decreased FcγRIIIa binding (60). The impaired FcγRIIIa binding and ADCC activity of IgG3 allotypes expressing a leucine at position 291 or a tryptophan at position 292 cannot be directly explained on the existing co-structures of IgG-Fc and FcγRIIIa since these residues are positioned outside the binding interface. We speculate that these residues change the conformation of the C’E loop, which affects the interaction of the receptor with the Fc of IgG3. Fucosylation of the IgG-N297 glycan has been suggested to hinder the conformational freedom of the IgG-glycan, sterically interfering with FcγRIII binding due to its unique glycosylation site at N162 not found in the other FcγR (61). A particular conformation of the C’E loop might also restrict the conformational freedom of FcγR in the recognition of the IgG Fc domain. Although neighboring residues 291 and 292 are both positioned outside the binding interface with FcγR, only the variation at position 292 significantly affected the affinity for FcγRIIIa in the classical SPR. In the cellular SPR set-up, however, both L291 and R292 expressing IgG3 allotypes were found to have a significantly lower binding to FcγRIIIa compared to other allotypes. The cellular SPR measurements resemble the observations in ADCC more closely, emphasizing the importance of measuring avidity of Fc – FcγR interactions. Compared to the other determinants (residue 292, 296), the influence of L291 on FcγRIIIa binding was largely amplified when avidity was taken into account. We hypothesize that the L291 has a different impact on the CH2 domain conformation in an antigen bound situation (cellular SPR) compared to an antigen unbound situation (SPR). Further studies are necessary to understand how the allotypic determinants proximal of the N297 glycan influence FcγRIIIa affinity and avidity mechanistically.

Several studies have found that the length and flexibility of an antibody influences FcγR binding and ADCC activity (62–65). However, there is no general consensus whether a short or a long hinge is beneficial for ADCC activity. We found that IgG3 antibodies with a short hinge, IgG3 allotype ^∗^04 (IGHG3^∗^04; 2 exons) and IgG3 with an IgG1 hinge (1 exon), showed the strongest ADCC capacity, which was not reflected by an increased affinity for the receptor FcγRIIIa. We hypothesize that a long hinge increases the distance between the target cell and the effector cell at the immunological synapse of Fc-FcγRIIIa interactions, thereby reducing ADCC efficiency. In line with this hypothesis, positioning the antigen further away from the target cell membrane was shown to reduce NK-cell mediated ADCC of target cells (66). Whether the hinge length enhances or reduces ADCC activity was found to be highly epitope dependent for anti-EGFR antibodies (65). Furthermore, in the context of HIV-1, IgG3 antibodies with a long hinge were more efficient in inducing phagocytosis and trogocytosis, but hinge length did not influence ADCC activity (52). This context-dependency of hinge length and ADCC activity is intriguing and warrants further investigation.

The short hinge of IgG3 allotype IGHG3^∗^04 and IgG1 antibodies might allow for a longer contact time between effector and target cell, which results in more efficient signaling and delivery of perforin and granzymes (4, 7). A short distance between effector and target cell possibly excludes some large membrane bound proteins from the immunological synapse. Indeed, this size-dependent segregation of the inhibitory phosphatase CD45 from the immunological synapse was found to regulate phagocytosis of target cells (67). The length of the hinge has also been described to influence antibody dependent complement activation on bacteria, emphasizing the broad spectrum of fc-effector functions that are affected by the hinge length of an IgG subclass or allotype (63, 64, 68, 69).

The polymorphic variants that are described for subclasses IgG1, IgG2, and IgG4 are defined by small amino acid variation, that did not affect FcγR binding and function. A limitation of this study is that despite lack of apparent FcγR-binding variation within IgG2 and IgG4 allotypes, we did not formally test this functionally in assays depending on FcγRI and FcγRIIa. A few amino acid variations between allotypes within the IgG3 subclass resulted in significant differences in FcγR binding and ADCC. This suggests that selective pressure on IgG3 effector function has specifically driven the emergence of polymorphic variants in the IgG3 heavy chain constant domain. IgG3 is known to be produced during early responses to viral pathogens and was found to be correlated with a lower infection risk in an HIV vaccine trial (70–72). Furthermore, antigen specific IgG3 antibodies were found to correlate with the control of chikungunya virus and long term protection against Malaria (73, 74). Whether early acquired anti-viral IgG3 antibodies are capable of controlling viral infections might be dependent on the Fc effector function of the IgG3 allotype. Therefore, it would be of interest to screen for IgG3 polymorphisms in cohort studies on infectious diseases and determine whether IgG3 allotype is linked to protection or control of various infectious diseases.

In summary, this work constitutes the first comprehensive exploration of ADCC activity of all known (at the initiation of the study) 27-human IgG allotypes. We identified determinants in the CH2 and hinge domain of the IgG3 allotypes that influence ADCC capacity by modulating the proximity between effector and target cells, but also by affecting FcγR affinity- and/or avidity. These findings were validated with four different panels of antibody with specificities relevant for alloimmune diseases but also tumor therapies. These findings might open new strategies in the development of therapeutic antibodies and shed light on the associations between IgG allotypes and infectious disease.

## Data Availability Statement

All datasets generated for this study are included in the article/Supplementary Material.

## Ethics Statement

Ethical review and approval was not required for the study on human participants in accordance with the local legislation and institutional requirements. The patients/participants provided their written informed consent to participate in this study.

## Author Contributions

ST, SL-T, and NS cloned the expression vectors encoding human anti-RhD and anti-TNP allotypes. ST and NS produced and isolated the human anti-RhD and anti-TNP IgG allotypes and mutants. JM designed anti-CD20 mutant hinge antibodies. ST and AB performed and analyzed the FcγR affinity and avidity measurements with SPR and cSPR, respectively. DF and TS analyzed the Fc glycosylation of the IgG allotypes with mass spectrometry in the lab of MW. ST performed and designed the ADCC assays with anti-TNP and anti-RhD antibodies. JM and MM performed the ADCC assays with anti-CD20 and anti-CD52 antibodies. GV, TR, AL, and JS designed the experiments and supervised the project. ST, AL, TR, and GV wrote the manuscript which was critically reviewed by all authors.

## Conflict of Interest

JM, MM, AL, and JS were employed by the company Genmab. The remaining authors declare that the research was conducted in the absence of any commercial or financial relationships that could be construed as a potential conflict of interest.
