# Supplementary material for: FcγR Binding and ADCC Activity of Human IgG Allotypes
Source: Front Immunol. 2020 May 6;11:740. doi: 10.3389/fimmu.2020.00740 (PMC7218058; doi:10.3389/fimmu.2020.00740)
Supplement: Supplementary file 1 [file Data_Sheet_1.pdf]

Supplementary material

FcγR binding and ADCC activity of human IgG allotypes

Steven W. de Taeye[1,2,*,], Arthur E.H. Bentlage[2], Mirjam M. Mebius[3], Joyce I. Meesters[3], Suzanne Lissenberg-Thunnissen[2], David Falck[4], Thomas Sénard[4], Nima Salehi[1], Manfred Wuhrer[4], Janine Schuurman[3], Aran F. Labrijn[3], Theo Rispens[1,†], Gestur Vidarsson[2,†]

[1] *Sanquin Research, Dept Immunopathology and Landsteiner Laboratory, Amsterdam UMC, University of Amsterdam, Amsterdam, The Netherlands*
[2] *Sanquin Research, Dept Immunohematology Experimental and Landsteiner Laboratory, Amsterdam UMC, University of Amsterdam, Amsterdam, The Netherlands*
[3] *Genmab, Utrecht, The Netherlands*
[4] *Center for Proteomics and Metabolomics, Leiden University Medical Center, Leiden, The Netherlands*

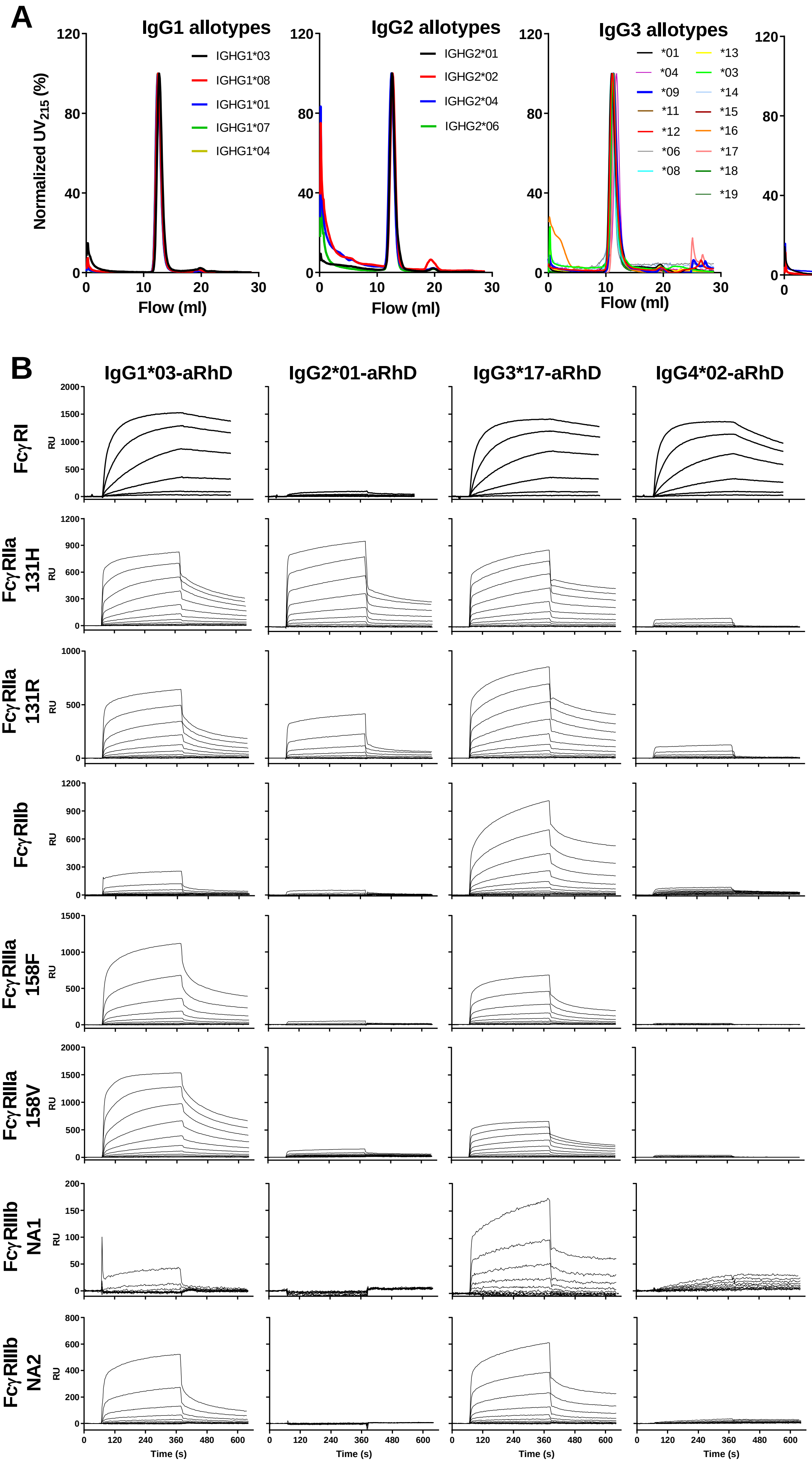

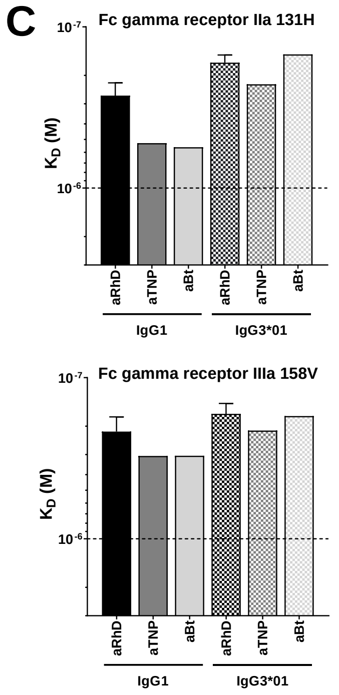

SI Figure 1: Characterization of oligomer formation and Fc gamma receptor binding of purified IgG allotypes

(**A**) Anti-RhD IgG allotypes (20 µg) were subjected to high-performance size exclusion chromatography (HP-SEC) on a superdex-200 column. Normalized UV215 elution profiles of all allotypes are depicted per subclass. (**B**) Binding of IgG allotypes to fc gamma receptors as determined by SPR. Individual sensorgrams of one allotype from each subclass (IGHG1*03, IGHG2*01, IGHG3*17 and IGHG4*02) to all FcγR are depicted. (**C**) Binding affinity of IgG1 and IgG3*01 with various specificities (aRhD, aTNP and aBiotin) to FcγRIIa 131H and FcγRIIIa 158V. Affinities for anti-RhD antibodies are identical to those plotted in figure 1. Binding affinities for aTNP and aBt antibodies were only measured once (n=1).

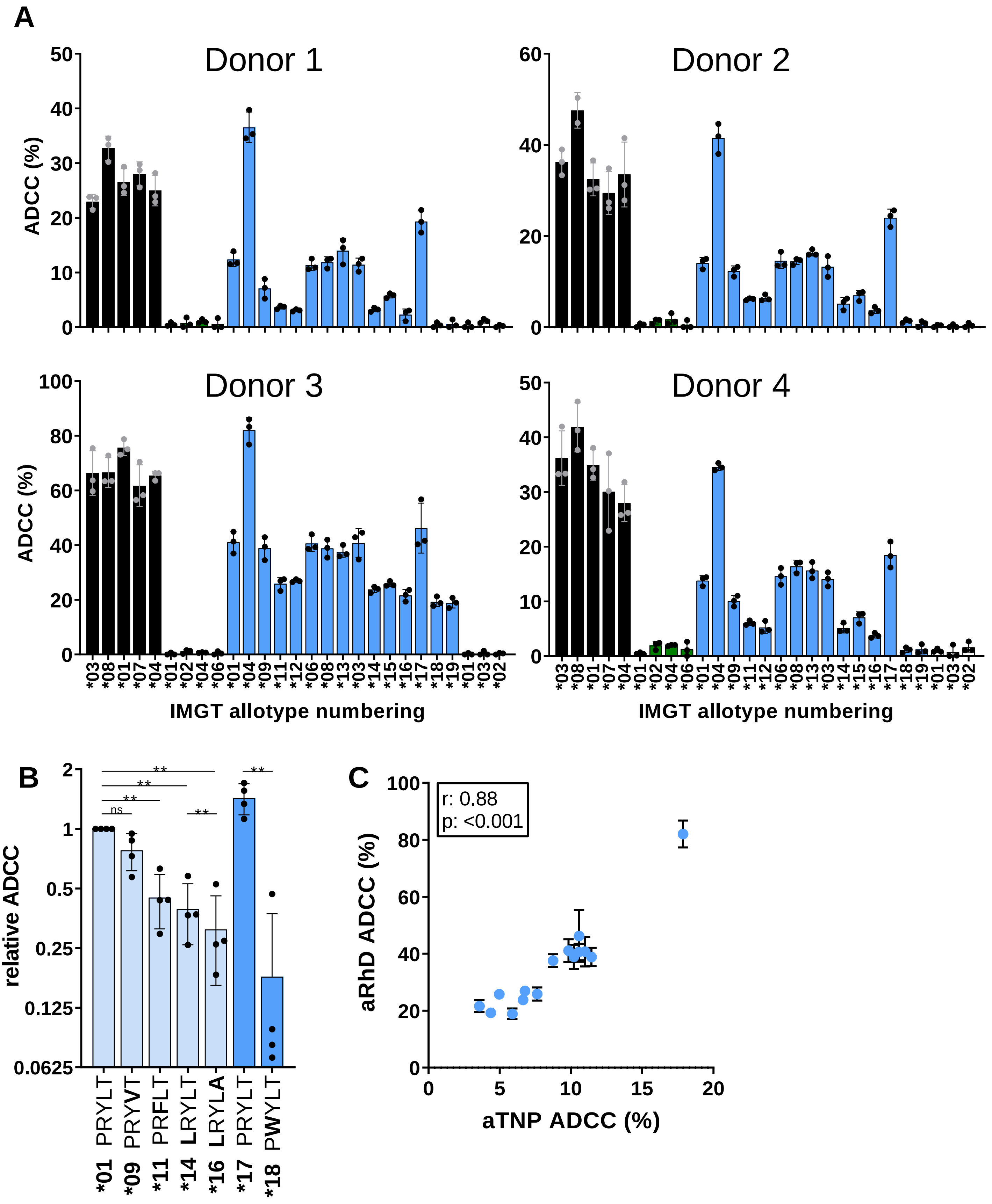

SI Figure 2: ADCC capacity of anti-RhD allotypes

(**A**) NK cell mediated ADCC capacity was assessed for all anti-RhD IgG allotypes with NK cells obtained from four individual donors. Measurements for each allotype in triplo are plotted as bar graphs, in which black bars represent IgG1 allotypes, green bars IgG2 allotypes, blue bars IgG3 allotypes and white bars IgG4 allotypes. (**B**) Relative ADCC of a selection of IgG3 allotypes to illustrate the influence of the CH2 domain on ADCC activity. The relative ADCC activity was calculated by comparing the mean ADCC activity in panel **A** with the mean ADCC activity of IgG3 allotype *01. On the y-axis the IgG3 allotype number is given together with the CH2 domain sequence. Variations in the CH2 domain compared to allotype *01 are indicated in bold. (**C**) Correlation plot of anti-RhD ADCC and anti-TNP ADCC of IgG3 allotypes (raw data in figure 2F-G). Correlation of the two parameters is determined with a non-parametric spearman correlation.

A

	CH1	Hinge				
	131	A	B	B	B	length
IgG3*01	C	+	+	+	+	62
IgG3*01-rch3	C	+	+	+	-	47
IgG3*04	C	+	+	-	-	32
IgG3*01-rch1A	C	+	-	-	-	17
IgG3*01-rch1B	C	-	+*	-	-	15
IgG3*01-h1	S	+	-	-	-	15
IgG1*03	S	+	-	-	-	15

* includes C219S to retain IgG3-like HC-LC pairing

B

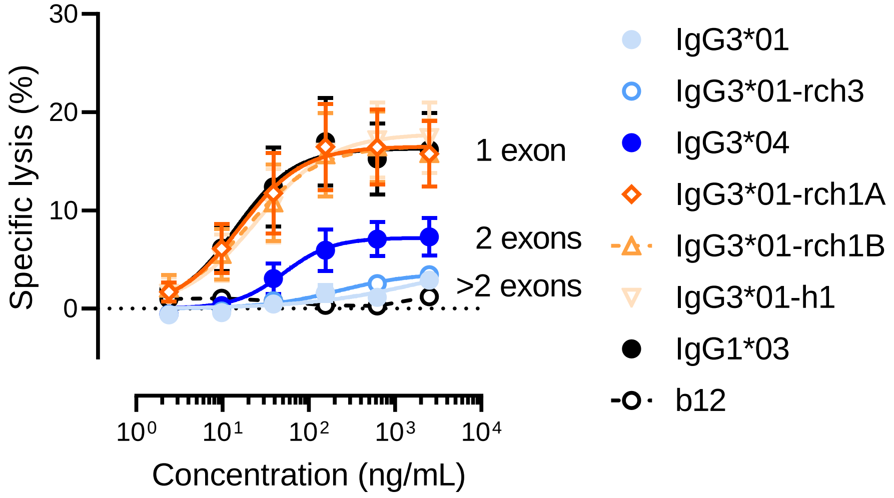

SI Figure 3: Influence of the hinge-length on IgG3-mediated ADCC

(**A**) Matched set of natural (IgG3*01 and IgG3*04) and IgG3*01 hinge mutants (containing deletions or substitutions of selected hinge exons) representing a range of hinge-lengths. (**B**) The capacity of CD20-specific IgG3 hinge-length variants to induce PBMC-mediated ADCC was assessed in vitro. Specific lysis of Raji cells was determined by subtracting mean background lysis, determined from control wells without antibody. Data represent mean ± SEM of n=6 donors (FcγRIIIa polymorphism genotypes unknown).

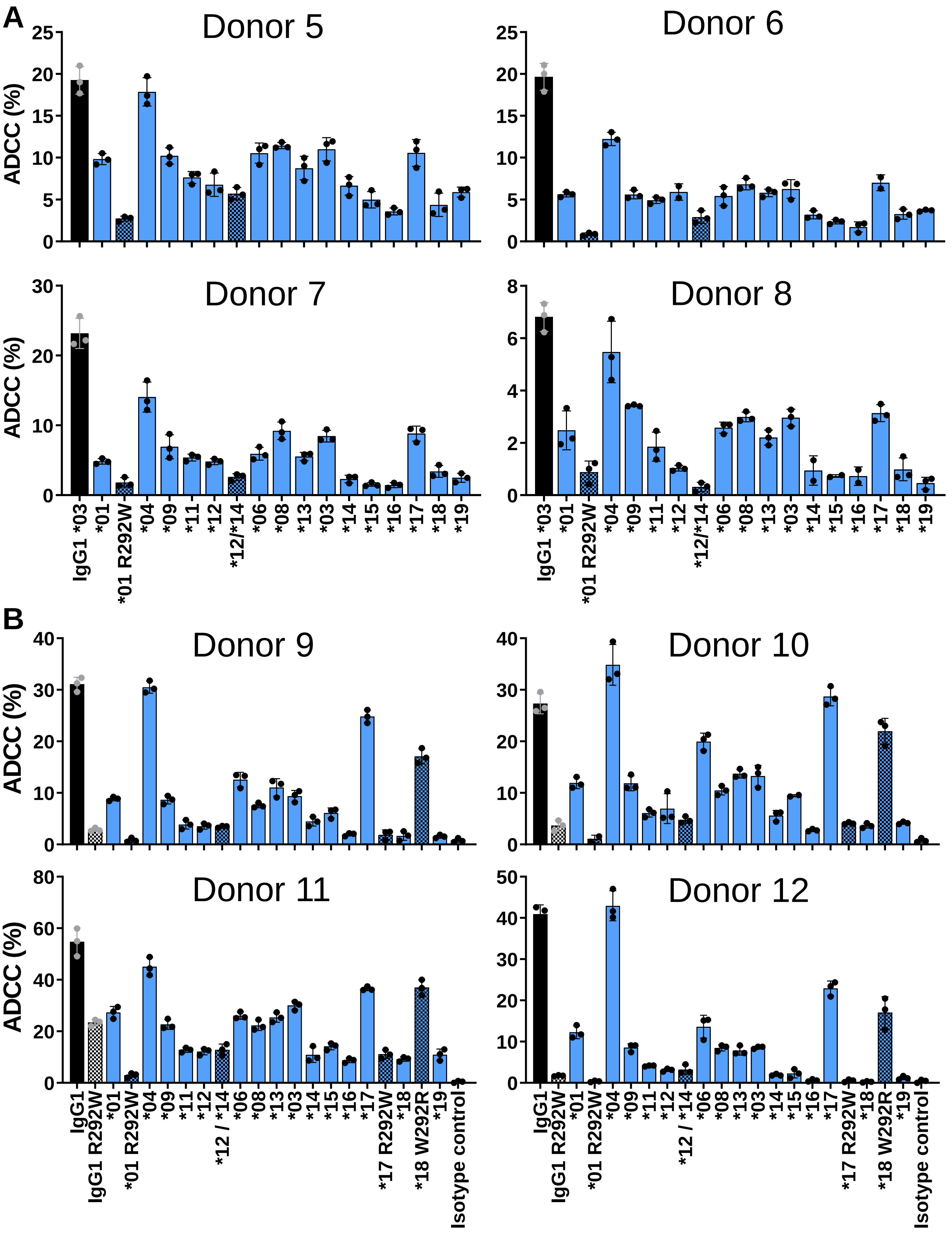

SI Figure 4: ADCC capacity of anti-TNP / anti-RhD allotypes and mutants

NK cell mediated ADCC capacity was assessed for all (**A**) anti-RhD and (**B**) anti-TNP IgG allotypes and mutants with NK cells isolated from four individual donors. Measurements for each antibody in triplo are plotted as bar graphs, in which black bars represent IgG1 allotypes and blue bars IgG3 allotypes. Mutant antibodies are indicated by checkered bars. The relative ADCC activity was calculated based on the mean of the triplo measurement. The relative ADCC data is presented in figure 3A-B, 4C-D and 5B-C.

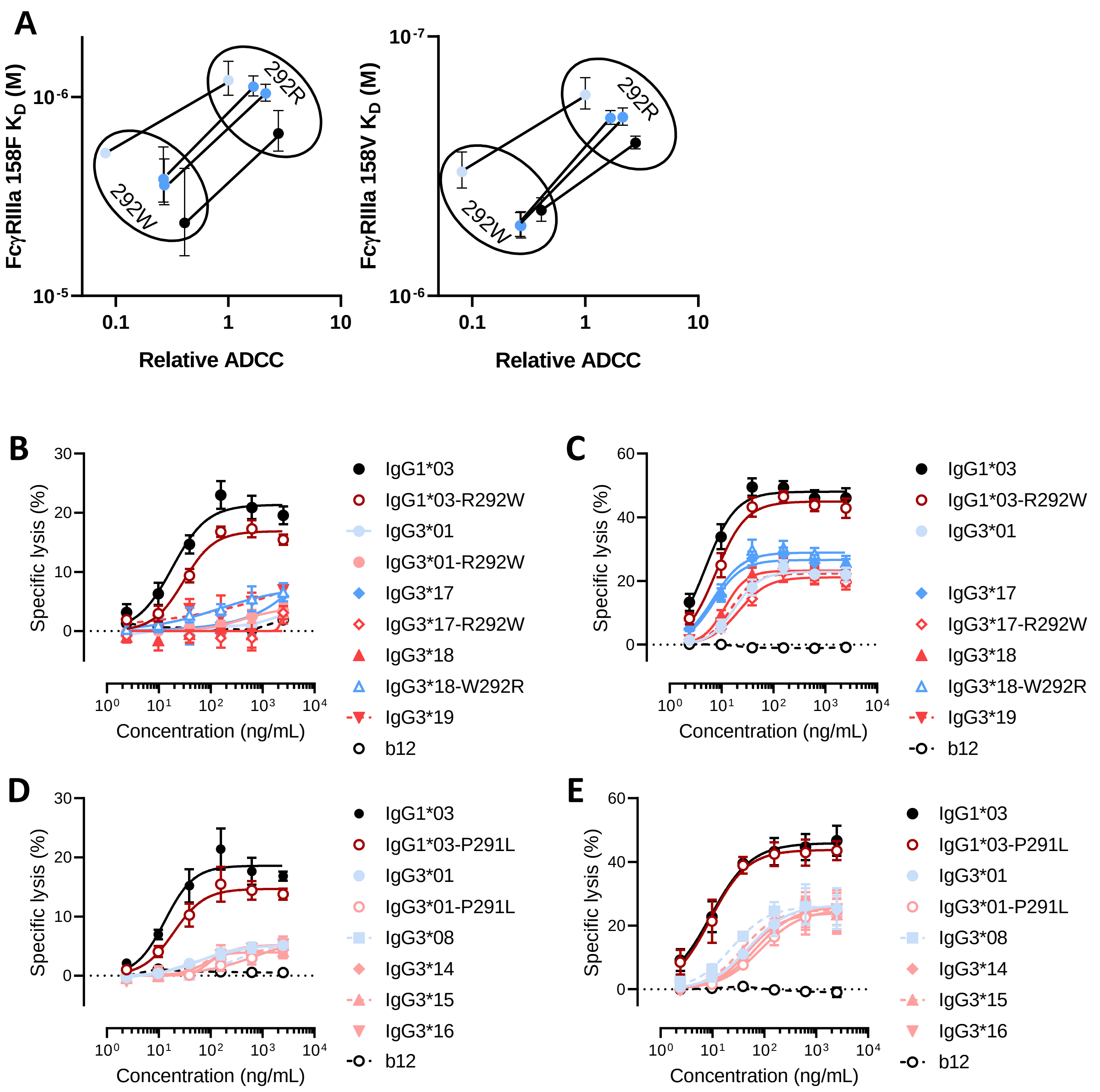

SI Figure 5: Influence of selected CH2 domain residues on IgG3-mediated ADCC

(**A**) Correlation plot of FcγRIIIa affinity (158F or 158V) and relative ADCC activity of 292-mutated anti-RhD antibodies. 292-mutant antibodies are connected with a black line to their parental antibodies (IgG3*01, *17, *18 and IgG1) and 292R and 292W expressing antibodies are encircled in the graph. Black dots represent IgG1, blue dots IgG3 antibodies with an intermediate hinge (47AA) and light blue dots represent IgG3 antibodies with a long hinge (62AA). The capacity of CD20-specific (**B,D**) and CD52-specifc (**C,E**) IgG1*03, indicated IgG3 allotypes and mutants to induce PBMC-mediated ADCC was assessed *in vitro*. Specific lysis of Raji (**B,D**) and Wien-133 (**C,E**) cells was determined by subtracting mean background lysis, determined from control wells without antibody. IgG3 allotypes and mutants are colored according to hinge length and residue, with long hinge in light colors, intermediate hinge length in regular colors, L291 or W292 in red(s) and P291 or R292 in blue(s). Data in graphs B, D and E represent mean ± SEM of n=3 donors, data in graph C represent mean ± SEM of at least n=6 donors.

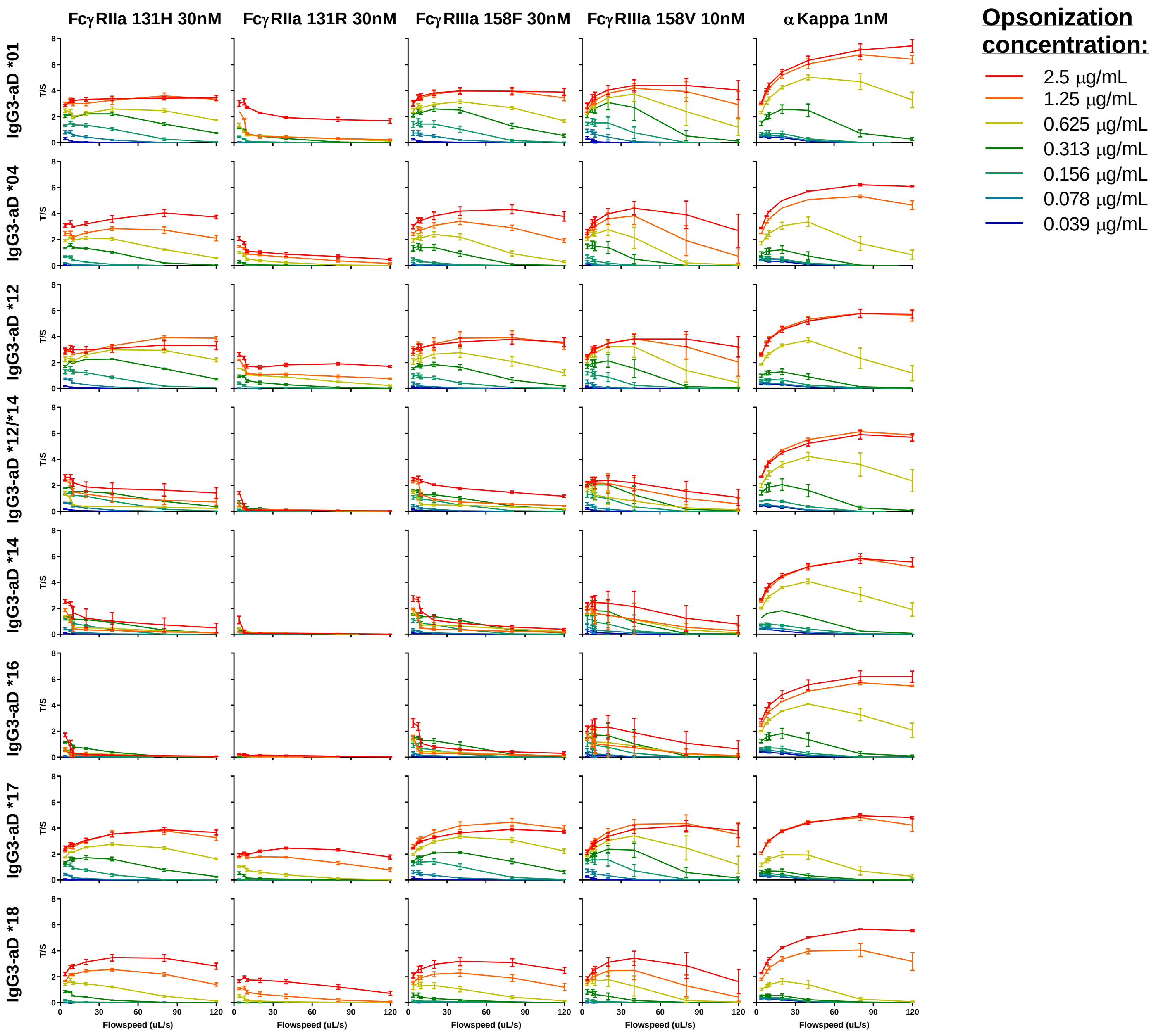

SI Figure 6: FcγR avidity measurements of IgG3 allotypes

Avidity measurements to FcγRIIa 131R, FcγRIIa 131H, FcγRIIIa 158F, FcγRIIIa 158V were determined for seven IgG3 allotypes (*01, *04, *12, *14, *16, *17, *18) and one IgG3 mutant expressing a L291 (*12/*14). Total / Sedimentation (T/S) RU ratios are plotted to indicate the binding strength of the opsonized RBCs to the FcγR after each increase in flow speed. Each color represents a particular level of opsonization, ranging from 2.5 µg/ml to 0.078 µg/ml. Binding strength to an anti-kappa nanobody was determined simultaneously. to confirm equal opsonization levels with each allotype. Error bars indicate SD of ≥3 independent measurements.

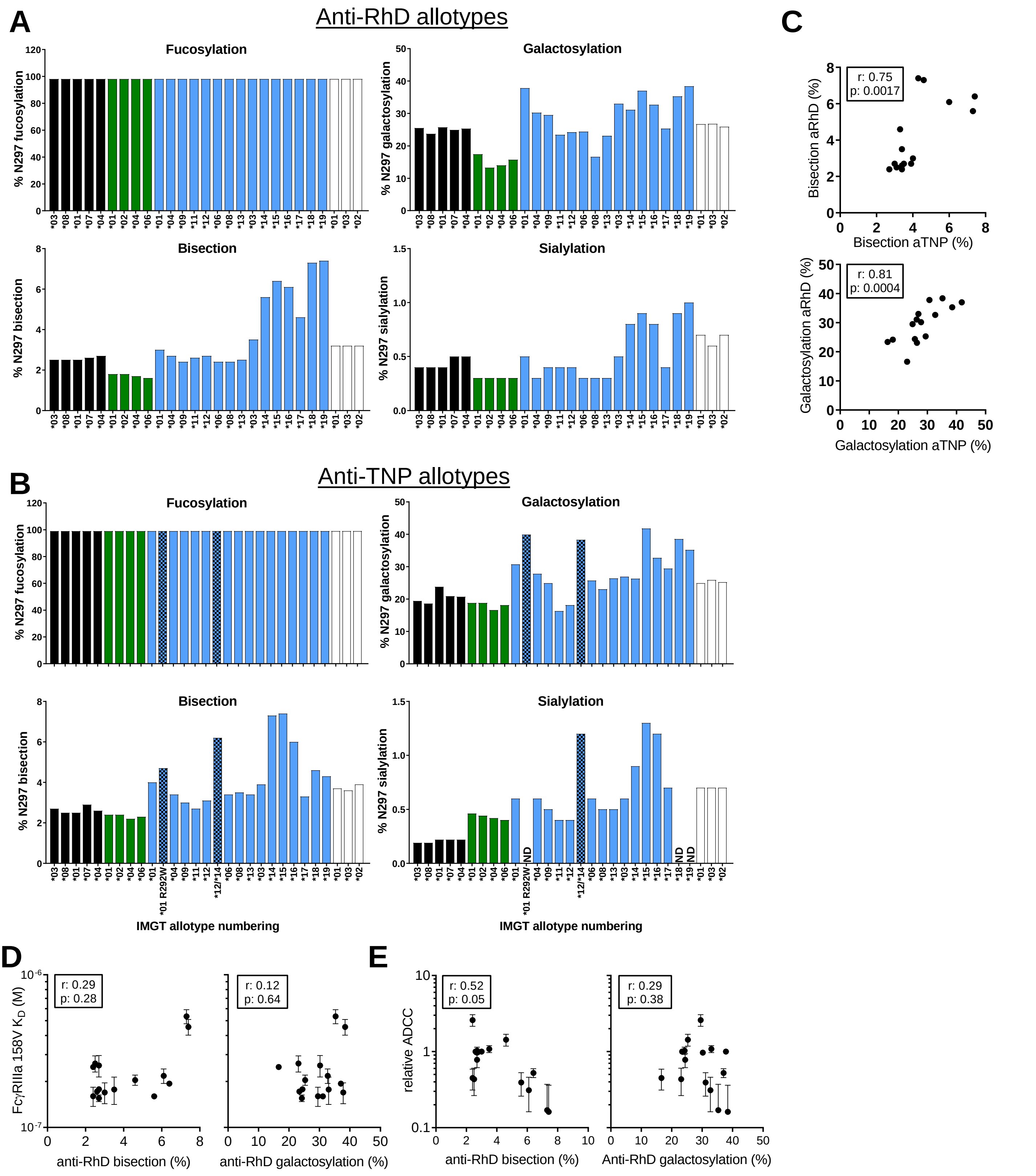

SI Figure 7: N297-glycosylation analysis of IgG allotypes

Glycosylation profile of the N297 Fc glycan of all (**A**) anti-RhD and (**B**) anti-TNP allotypes as determined by mass spectrometry. The glycosylation profile is subdivided in four panels showing the percentage of fucosylation, galactosylation, bisection and sialylation, which was calculated from the different glycopeptide species that were identified. Quantification of low abundant afucosylated glycopeptides was difficult, therefore an estimation of >98% fucosylation is depicted for all allotypes. Black bars represent IgG1 allotypes, green bars IgG2 allotypes, blue bars IgG3 allotypes and white bars IgG4 allotypes. Allotypes IGHG3*18, IGHG3*19 and mutant IGHG3*01 R292W express a tryptophan instead of an arginine at position 292, which changes the length of the peptide containing the N-linked glycan after trypsine digestion. The analysis is not optimized for these allotypes with a longer tryptic glycopeptide and therefore the data is of less quality or percentages could not be determined (N.D.). (**C**) Correlation plot showing bisection or galactosylation levels of IgG3 anti-RhD and anti-TNP allotypes (**D**) FcγRIIIa affinity of IgG3 anti-RhD allotypes as measured by SPR or (**E**) relative ADCC capacity is plotted against the percentage of N297 galactosylation or bisection. Correlation of the two parameters is determined with a non-parametric spearman correlation.
